# RagC and Map4K3 deficiency in high-grade gliomas drives proliferation and modulates mTORC1-dependent cellular functions

**DOI:** 10.1093/jnen/nlag010

**Published:** 2026-03-22

**Authors:** Julian Kahr, Roberto Diaz-Peregrino, Ibrahim E Sandalcioglu, Peter John, Christian Mawrin

**Affiliations:** Department of Neuropathology, Otto-von-Guericke University, Magdeburg, Germany; Department of Neurosurgery, University Hospital Jena, Jena, Germany; Department of Neuropathology, Otto-von-Guericke University, Magdeburg, Germany; Department of Neurosurgery, Otto-von-Guericke University, Magdeburg, Germany; Department of Neuropathology, Otto-von-Guericke University, Magdeburg, Germany; Department of Neuropathology, Otto-von-Guericke University, Magdeburg, Germany

**Keywords:** amino acid sensor, autophagy, glioma, Map4K3, RagC

## Abstract

Cellular growth and homeostasis via amino acid-responsive pathways are mediated by the mTOR signaling pathway. Rag GTPases and Map4K3 modify mTOR signaling as amino acid sensors. Altered mTOR signaling in relation to amino acid sensors might represent factors that modify proliferation and treatment responses in astrocytic tumors. To investigate this hypothesis, RagC and Map4K3 expression was studied in human gliomas, glioma cells (U87MG/U138MG), and nonglial cells (MCF-7, IOMM-Lee). RagC and Map4K3 knockout in glioma cells was generated using CRISPR-Cas and shRNA. High-grade astrocytomas had significantly reduced immunoreactivity for RagC and Map4K3 compared to low-grade astrocytomas. RagC- and Map4K3-deficient glioma cells had significantly increased proliferation and showed altered morphology and motility. Induced amino acid deficiency (leucine deprivation) reduced proliferation in Map4K3- but not in RagC-deficient cells. mTOR signaling in RagC- and Map4K3-deficient U87 cells was altered with increased phosphorylation of p70S6K and increased expression of RagD and transcription factor EB. In this context, uncoupled, exaggerated autophagy occurred in Map4K3-deficient U87 cells. In contrast, RagC-deficient U87 cells showed increased senescence but no autophagy induction. These data show that losses of RagC and Map4K3 in malignant gliomas have proliferation-inducing effects and differentially modulate key mTOR signaling-dependent cellular mechanisms.

## INTRODUCTION

Gliomas are primary brain tumors that are categorized based on their World Health Organization (WHO) grade, ranging from grade 1 to grade 4.^1^ WHO grade 1 gliomas are considered to be the least aggressive and have a better prognosis, while WHO grade 4 gliomas are highly malignant and have poor prognoses.^2^^,^^3^ Treatment options for gliomas depend on several factors, including the type, grade, and location of the tumor. Treatment may involve surgical resection of the tumor, radiation therapy, and chemotherapy. However, overall, treatment options are still limited.^3^ This is partly based on an incomplete understanding of the biological mechanisms acting in gliomas.

High-grade gliomas are notorious for their ability to grow and divide uncontrollably. This uncontrolled growth requires vast amounts of nutrients including amino acids.^4^ Consequently, glioma cells have developed specialized mechanisms to sense and respond to changes in amino acid availability.^5^ An increased uptake of amino acids, especially branched-chain amino acids such as leucine, provides the necessary nutrients for glioma cells to sustain their growth and proliferation.^6^ Glioma cells exhibit alterations in leucine catabolism, resulting in the production of metabolites that can promote tumor growth and progression.^7^

A crucial pathway for cells to respond to changes in amino acid availability is the mTOR signaling pathway.^8^ This pathway regulates cell growth and metabolism in response to changes in amino acid availability. The mTOR complex 1 (mTORC1) is a key player, particularly for leucine.^9^^,^^10^ Amino acids directly stimulate mTORC1-dependent phosphorylation of ribosomal protein S6 kinase 1 (S6K1) and eukaryotic initiation factor 4E-binding protein 1 (4E-BP1), whereas amino acid deprivation results in rapid dephosphorylation.^11^ These effects are mediated by the amino acid sensors Ras-related GTP binding (RagC) and mitogen-activated protein kinase kinase kinase kinase 3 (Map4K3), which are located upstream of mTORC1.^12^^,^^13^

RagC is one of the Rag GTPases that functions as a critical amino acid sensor within the mTOR pathway.^12^ The Rag GTPases, composed of RagA and RagB (forming Rag heterodimers), and RagC and RagD (forming Rag heterodimers), play pivotal roles in regulating the localization and activation of the mTORC1 complex in response to changes in amino acid availability.^14^^,^^15^ The Rag GTPases act as molecular switches that cycle between an active GTP-bound state and an inactive GDP-bound state.^16^

In its GTP-bound form, RagC acts as a scaffolding platform that anchors the Rag heterodimers to the lysosomal surface, where mTORC1 is activated.^15^ As a result, mTORC1 can initiate downstream processes that lead to increased protein synthesis and cell growth and modulate autophagy depending on the nutrients by binding the RagGTPases with transcription factor EB (TFEB).^8^^,^^17^^,^^18^ Transcription factor EB is a key regulator of the cellular process known as autophagy, which is responsible for the degradation and recycling of cellular components.^19^

Map4K3 is a Ste20-familiy member and involved in wide range of cellular processes.^20^ Studies in *Drosophila* have shown that Map4K3 is essential for mTORC1 activity and have demonstrated that MAP4K3 depletion or inhibition leads to decreased mTORC1 activity and impaired cell growth and proliferation.^13^^,^^21^^,^^22^ The MAP4K3 activation by amino acids occurs before S6K phosphorylation and is not inhibited by the mTOR inhibitor rapamycin, indicating that MAP4K3 can be placed upstream of mTORC1.^23^ Furthermore, by phosphorylating and activating the Rag GTPases, Map4K3 may contribute to the regulation of mTORC1 activation in response to amino acid availability. Investigations related to autophagy, a key cellular mechanism associated with amino acid turnover,^24^ have shown that MAP4K3 can phosphorylate and activate the Rag GTPases, specifically RagC and RagD, leading to the recruitment of mTORC1 to the lysosomal surface and subsequent mTORC1 activation.^25^

The roles of RagC and Map4K3 in gliomas have not been studied in detail so far. Alterations of amino acid sensors may contribute to the dysregulation of mTORC1 signaling commonly observed in different pathological conditions.^26–28^ In brain tumors, low-grade astrocytomas occurring in the context of tuberous sclerosis (TSC) respond well to mTOR inhibition by Everolimus, while clinical studies addressing mTOR inhibition in high-grade gliomas failed.^29^^,^^30^ One reason for the latter might be a different function of mTOR outside the TSC-driven tumor condition.

## METHODS

### Cell culture and reagents

Cells were cultured in 250 mL plastic flasks T75 (Cellstar, Greiner Bio-One, Kremsmuenster, Austria) with Dulbecco’s modified Eagle’s medium ([DMEM] 4.5 g/L glucose, PAN Biotech, Aidenbach, Germany), supplemented with 10% fetal calf serum (FCS) and 1% penicillin/streptomycin (PAN Biotech). The flasks were incubated at 37 °C and 5% CO_2_. For various experiments, cells were seeded in appropriate density into plates (see below). Leucine restriction was performed using DMEM without leucine from PAN Biotech containing dialyzed FCS from Thermo Scientific (Waltham, MA, United States). For this purpose, cells were treated for 4 h and restimulated by adding DMEM with 10% FCS for 1 h. For autophagy flux tracking, we used NH_4_Cl at a concentration of 20 mM for 4 h.

### Cell lines

Malignant human U87MG, U138MG glioma cell lines and malignant human breast cancer cell line MCF-7 were purchased from ATCC (American Type Culture Collection; Manassas, VA, United States). Malignant human IOMM-Lee meningioma cells were kindly provided by David H. Gutmann, Washington University.

### Lentiviral transfection

The following lentiviral plasmids, harbored in *Escherichia coli* bacteria, were acquired from VectorBuilder (Chicago, IL, United States) for later transfection of human U87MG or U138MG glioma cells or of human MCF-7 breast cancer cells: (1) a human CRISPR-Cas9 construct containing 2 RRAGC gRNAs and puromycin resistance; (2) the same CRISPR-Cas9 construct, but containing 2 MAP4K3 gRNAs and puromycin resistance; and (3) the same CRISPR-Cas9 construct, but containing 2 scrambled small RNAs replacing the 2 specific gRNAs (control). For later transfection of human IOMM-Lee malignant meningioma cells, the same MAP4K3, RagC, and scrambled CRISPR-CAS plasmids were used, but the gene mediating puromycin resistance was replaced by the selectable marker blasticidin resistance. All lentiviral plasmids were packaged with psPAX2 and pMD2.G into lentiviral particles using HEK 293 T cells. Collected supernatants were used to transfect the target cells, which were thereafter selected over 2 weeks with 3 µg/mL puromycin (AppliChem, Darmstadt, Germany), or 20 µg/mL blasticidin (Corning, Corning, NY, United States), respectively.

For generation of MAP4K3-knockdown and RagC-knockdown U87MG cells, HEK293T cells (DSMZ, Braunschweig) were transfected using FuGene HD transfection reagent (Promega, Mannheim, Germany) with pLV[shRNA]-Puro-U6>hMAP4K3[shRNA#1] or pLV[shRNA]-Puro-U6> hRAGC[shRNA#4] (VectorBuilder) in combination with lentiviral packaging plasmid mix pC-Pack 2 (Cellecta, Mountain View, CA, United States) as reported.^31^ Supernatants were harvested after 24 and 48 h, filtered, and used for infection of U87MG cells. Infected cells were finally selected with puromycin.

### Map4K3 and RagC knockout screening

After antibiotic selection, a Western blot was performed to determine the potency of the Map4K3 and RagC knockout (KO). Subsequently, clones of these Map4K3- or RagC-CRISPR transfected and puromycin- or blasticidin-resistant cells were isolated, expanded, and finally tested a second time by Western blot to check clones with complete Map4K3- or RagC-KO compared with SCR controls. One each of Map4K3- or RagC-KO clones without residual expression of the protein were used for the assays.

### Tumor material and immunohistochemical studies

Human tumor material was used for immunohistochemical RagC detection and displayed in 4 representative images. The antibody RagC D31G9 (#5466, Cell Signaling Technologies, Boston, MA, United States) was used at a dilution of 1:200.

### mRNA expression

To investigate the expression of RagC, Map4K3, and Vps34 in gliomas of different WHO grades, mRNA was collected from the tumor samples according to common procedures and analyzed by real-time PCR. The results were normalized for β2-microglobulin. A total of 36 diffuse astrocytomas of different WHO grades according to the 2021 WHO classification were analyzed (6 grade 2 IDH-mutant astrocytomas, 7 grade 3 IDH-mutant astrocytomas, 23 glioblastoma grade 4).

### Western blotting

Cells from T25 flasks were lysed in 1 mL Pierce radioimmunoprecipitation assay Buffer (Thermo Scientific), containing 10 µL sodium vanadate (100 mM), 10 µL protease inhibitor cocktail, and 1 µL dithiothreritol (1 mM). Supernatant obtained after cell lysis was mixed with reducing SDS-samples buffer (Sigma Aldrich, Merck, Darmstadt, Germany) and boiled. Samples were subjected to SDS-PAGE in 12.5% polyacrylamide gels and electro-transferred to the nitrocellulose membrane. Membranes were blocked with 5% nonfat milk or 5% bovine serum albumin (BSA) in TBS-Tween buffer, depending on the antibody supplier’s suggestions. Membranes were then probed with primary antibodies at 4 °C overnight in the same solution, washed and then incubated with peroxidase-conjugated secondary antibodies. Chemiluminescence images were visualized by a camera system (Fusion F×7, Peqlab Biotechnologies, Erlangen, Germany). Densitometric measurements of the antibodies listed below were performed using NIH ImageJ and each was appropriately set in relation to ß-actin.

Antibodies against the following proteins were obtained from Cell Signaling Technology: Akt1 (#2938) p-Akt S473 (#4060) 1/2000, p-Akt T308 (#9275) 1/1000, 4E-BP1 (#9452) 1/1000, p-4E-BP1 (#9459) 1/1000, LC3A (#4599) 1/1000, Map4K3 (#92427) 1/1000, p44/42 MAPK (#9102) 1/1000, mTOR (#2972) 1/1000, p-mTOR (#2971) 1/1000, RagA (#4357) 1/1000, RagC (#3360) 1/1000, RagD (#4470) 1/1000, p70S6K (#9202) 1/1000, p-p70S6K (#9206) 1/1000, TFEB (#37785) 1/1000. β-Actin antibody was acquired from Sigma Aldrich. Secondary antibodies were purchased from Cell Signaling Technology.

### Phalloidin staining

On sterile coverslips located inside a 6-well plate, 5000 cells were cultured in 2 mL standard DMEM. The cells were incubated for 16 h. Each coverslip was washed with phosphate-buffered saline (PBS) and fixed with 250 µL 4% paraformaldehyde/PBS. The coverslips were rewashed for 3 times. Then, Triton X-100 (Sigma Aldrich) was dropped onto each coverslip to permeabilize the cells, followed by 3 more washes. The coverslips were blocked with 300 µL 1% BSA/PBS and incubated for 30 min at 4 °C. The cells were then stained with phalloidin. Coverslips were incubated in 200 µL Phalloidin Alexa Fluor 546 (Invitrogen, Thermo Fisher, dilution 1:40; stock solution dissolved in PBS) for 30 min. Unbound dye was removed with PBS, and the coverslips were dried. Finally, the coverslips were mounted on glass slides. Afterward, a Keyence BZ-9000 confocal microscope (Osaka, Japan) was used to capture images of cells of each genotype.

The camera captured 10 cells for each genotype. The associated software was used to automatically calculate cell area (µm^2^) and circularity (from 0 to 1; 0 means completely rounded and 1 completely elongated).

### Cell viability assays

Cell viability was assessed with the colorimetric assay CCK-8 (MedChemExpress, Monmouth Junction, NJ, United States), which utilizes the reduction of a tetrazolium compound (WST-8) to a water-soluble formazan by dehydrogenases of living cells. Four thousand cells (U87, U138) or 2000 cells (MCF-7, IOMM-Lee) were seeded in 96-well plates and allowed to settle down for at least 24 h. Afterward, the cells were cultured for 48 h in full medium or leucine-free medium, as depicted in the figures. Thereafter, tetrazolium incubation was performed according to the manufacturer’s instructions and the plates were read in a NanoQuant infinite M200 ELISA reader (Tecan, Maennedorf, Switzerland) at 450 nm using Magellan 7.2 software.

ATP was quantified in cell cultures with 2000 cells in 100 μL medium per well. The cells were cultivated in a 96-well microplate (Greiner bio-one) for 48 h in standard DMEM. Then, 100 µL of CellTiter-Glo 2.0 reagent (Promega, Madison, WI, USA) was added to each well. The mixture was blended for 2 min on the rocking platform to induce cell lysis. The plate was incubated for 10 min to stabilize the luminescent signal. Finally, the emitted light was measured with the luminometer (Turner designs TD 20/20, San Jose, CA, United States). BrdU assay was performed using a 5-Bromo-2′-Desoxy-Uridine Kit (Roche Applied Science, Mannheim, Germany). Five thousand cells per well were seeded into a 96-well plate (Sarstedt, Nümbrecht, Germany) and incubated at 37 °C, 5% CO_2_. After 48 h, cells were stained according to manufacturer’s information and analyzed using Tecan-Reader Infinite 200.

Trypan blue is a stain recommended for dye exclusion procedures for viable cell counting. Thirty-two thousand cells were incubated per well of a 48-well plate (Thermo Scientific) for 48 h in standard DMEM. Afterward, cells were resuspended with 20 µL trypan blue staining (Sigma Aldrich) and was mixed with 20 µL cell suspension. Living cells were counted using a Neubauer chamber (Marienfeld Superior, Lauda-Koenigshofen, Germany).

### Senescence assay

For identifying senescent cells, 100 000 cells per well were seeded in 6-well plates and the Promega Senescence Kit was used according to the “manufacturer’s” protocol. Plates were incubated overnight at 37 °C and covered by Parafilm in a reduced CO_2_ environment. Subsequently, 5 fields of the blue-stained cells were imaged and counted using a Keyence BZ-9000 confocal microscope, and recorded as a proportion of all cells in each field.

### Live cell imaging

Using a 60-mm cell dish, 50 000 cells were seeded for at least 24 h in DMEM. Subsequently, cells were observed under a Keyence BZ-9000 confocal microscope in a chamber system (Tokai Hit INU-KI3-F1) under 5% CO_2_ influx and a temperature of 37 °C for 4 h. An image was generated every 5 min, which was overlaid to a video at the end using NIH ImageJ. For each genotype, 20 cells were used and followed for 4 h.

### Statistical analysis

GraphPad Prism 8.0.1 was utilized for statistical analysis and plot creation. Results were expressed as plots, in which bars and error bars represent mean and SD, respectively. The *P*-values are indicated as n.s., *P ≤ .05; **P ≤.01; ***P ≤ .001. For cell morphology, comparison among U87 SCR control, RagC-KO, and Map4K3-KO cells, a *t*-test with Welch correction was computed. Two-way ANOVA with Bonferroni multiple comparison posttest was performed to compare U87 Map4K3-KO, RagC-KO, and SCR control cells performance in vitro. t-Test with Welch correction was used to compare U87-deficient RagC or Map4K3 cells performance during in vitro assays under leucine and leucine-free conditions.

## RESULTS

### Reduced expression of RagC and Map4K3 in high-grade gliomas

**Figure 1.**
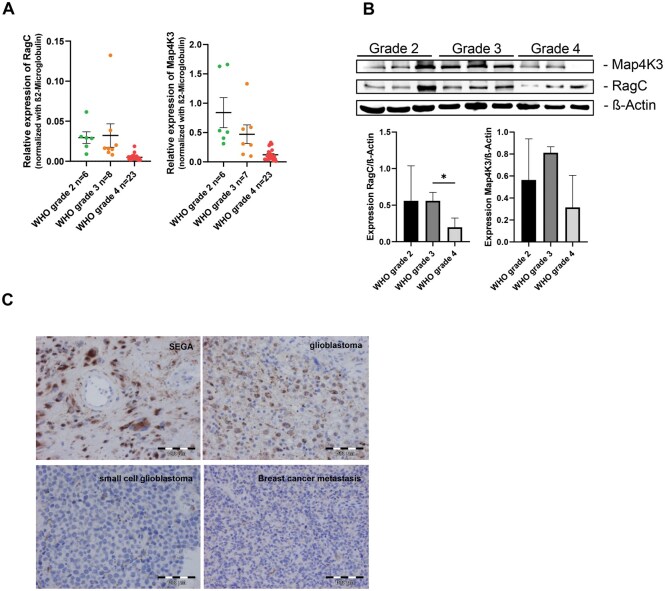
Expression of RagC and Map4K3 in gliomas. (A) Relative expression of RagC or Map4K3 in samples from WHO grade 2-4 gliomas analyzed by PCR. (B) Expression of RagC and Map4K3 in samples for WHO grade 2-4 gliomas via Western blot analysis. Immunoblotting of β-actin served as loading control. *n* = 3 for each WHO grade glioma, error bars = SD, *P < 05. (C) Examples of immunoexpression of RagC in samples of human tumors including subependymal giant cell astrocytoma (SEGA), glioblastoma, small cell glioblastoma, and metastasized breast cancer. Abbreviation: WHO = World Health Organization.

To answer the question whether the 2 amino acid sensors RagC and Map4K3 are differentially expressed between astrocytomas of different WHO grade, samples of diffuse astrocytoma, *IDH*-mutated grades 2 and 3 and glioblastoma with *IDH* wild-type were analyzed by real-time PCR for mRNA expression of RagC and Map4K3. As shown in Figure 1A, mRNA expression for each sensor is significantly reduced in glioblastoma samples. To confirm these findings, protein expression was examined and showed again a decreased expression in glioblastoma (Figure 1B). Figure 1C shows representative images of RagC immunoexpression in human gliomas and breast cancer metastasis. Interestingly, strong expression of RagC was observed in a circumscribed CNS-WHO grade 1 tumor (subependymal giant cell astrocytoma), which is known to have high mTOR activity, while expression in glioblastoma and breast cancer metastasis was reduced. These data suggest that highly proliferative tumors have unexpectedly reduced availability of amino acid sensors.

### Map4K3 and RagC deficiency in U87MG cells causes changes in proliferation, migration, and motility

**Figure 2.**
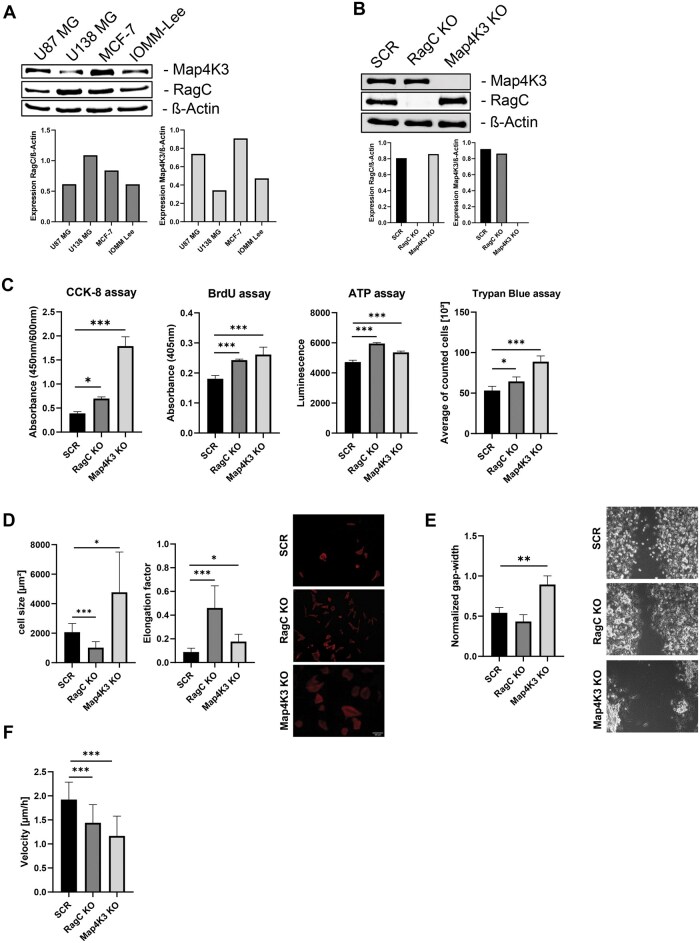
Proliferation, migration, and motility in Map4K3- and RagC-deficient U87MG cells. (A) Expression of RagC and Map4K3 in U87MG, U138MG, MCF-1, and IOMM-Lee cells. (B) Knockdown of RagC and Map4K3 in U87 CRISPR control (SCR), U87 CRISPR knockout RagC-KO, and Map4K3-KO cells. (C) CCK-8 BrdU, ATP, and trypan blue assays of SCR control, RagC-KO, and Map4K3-KO U87 glioma cells. (D) Cell size and circularity features of U87 SCR control, RagC-KO, and Map4K3-KO cells were computed. Additionally, pictures of U87 SCR, RagC-KO, and Map4K3-KO cells stained with phalloidin under the confocal microscope. *n* > 20 cells per genotype. (E) Photographic representation of the gap width after 6 h for U87 SCR, RagC-KO, and Map4K3-KO. The diagram shows the gap width after 6 h normalized to the initial gap width. (F) Observation of U87 SCR, RagC-KO and Map4K3-KO cells over 4 h with live cell imaging and tracking of 10 cells each. *P < .05, **P < .01, ***P < .001, error bars = SD.

Next, we tested expression in various glial and nonglial cell lines. As shown in Figure 2A, RagC and Map4K3 were clearly expressed in U87MG, U138MG, MCF-7, and IOMM-Lee cells, providing suitable models to knock down RagC and Map4K3 (Figure 2B and Figure S1C). By analyzing the proliferative potential of RagC or Map4K3 knockout cells, we observed a significant increase of proliferation in RagC-KO and Map4K3-KO U87 glioma cells within 48 h (Figure 2C). This finding was supported by similar effects in BrdU, ATP, and trypan blue assays, respectively. Additionally, to avoid the condition of metabolic stress, we directly counted cells by trypan blue staining and found again a significant increase in cell proliferation following knockout of both amino acid sensors (Figure 2C). Of note, initial experiments using with shRNA-induced knockdown of RagC and Map4K3 in U87MG cells showed a significant increase in proliferation for RagC but not for Map4K3 in ATP assay as well (Figure S2A and B).

To address the question of whether these effects are glioma-specific, we analyzed RagC- or Map4K3-deficient MCF-7 and IOMM-Lee cells by CCK-8 assay (Figure S2C). Interestingly, in MCF-7 and IOMM-Lee cells only RagC loss resulted in increased proliferation, whereas there was a significant decrease in proliferation in Map4K3-deficient MCF-7 cells and no significant decrease in proliferation in Map4K3-deficient IOMM-Lee cells.

Both mTORC1 and mTORC2 have been repeatedly shown to be involved in cell morphology and motility, including of cancer cells.^32–34^ Therefore, we wondered if modification of amino acid transporters involved in the regulation of mTOR function by downregulation of RagC and Map4K3 affects meningioma cell shape and motility.

Further investigations in cell morphology differences were investigated using phalloidin staining, as shown in Figure 2D. RagC-KO U87 cells were found to exhibit significant elongation associated with reduced cell size, while Map4K3-KO U87 cells exhibited an intriguing swollen morphology with increased cell size compared to SCR U87 controls. To assess the motility of RagC- or Map4K3-deficient U87 cells, we used a gap (Figure 2E). Here, we observed a significant difference between Map4K3-KO U87 cells and SCR U87 controls, with a lack of tendency to gap closure, in addition to a clustered cell network. In contrast, no significant difference in gap closure was observed for U87 RagC-KO cells. To further elucidate this finding, cells were subjected to live cell imaging (Figure 2F). Here, a strikingly reduced motility of Map4K3-KO U87 cells and a moderate motility reduction in RagC-KO U87 cells was observed. Visualization of the distance covered by the individual cells is shown in Figure S2E.

### RagC- and Map4K3-deficient glioma cells show increased mTORC1 activity

**Figure 3.**
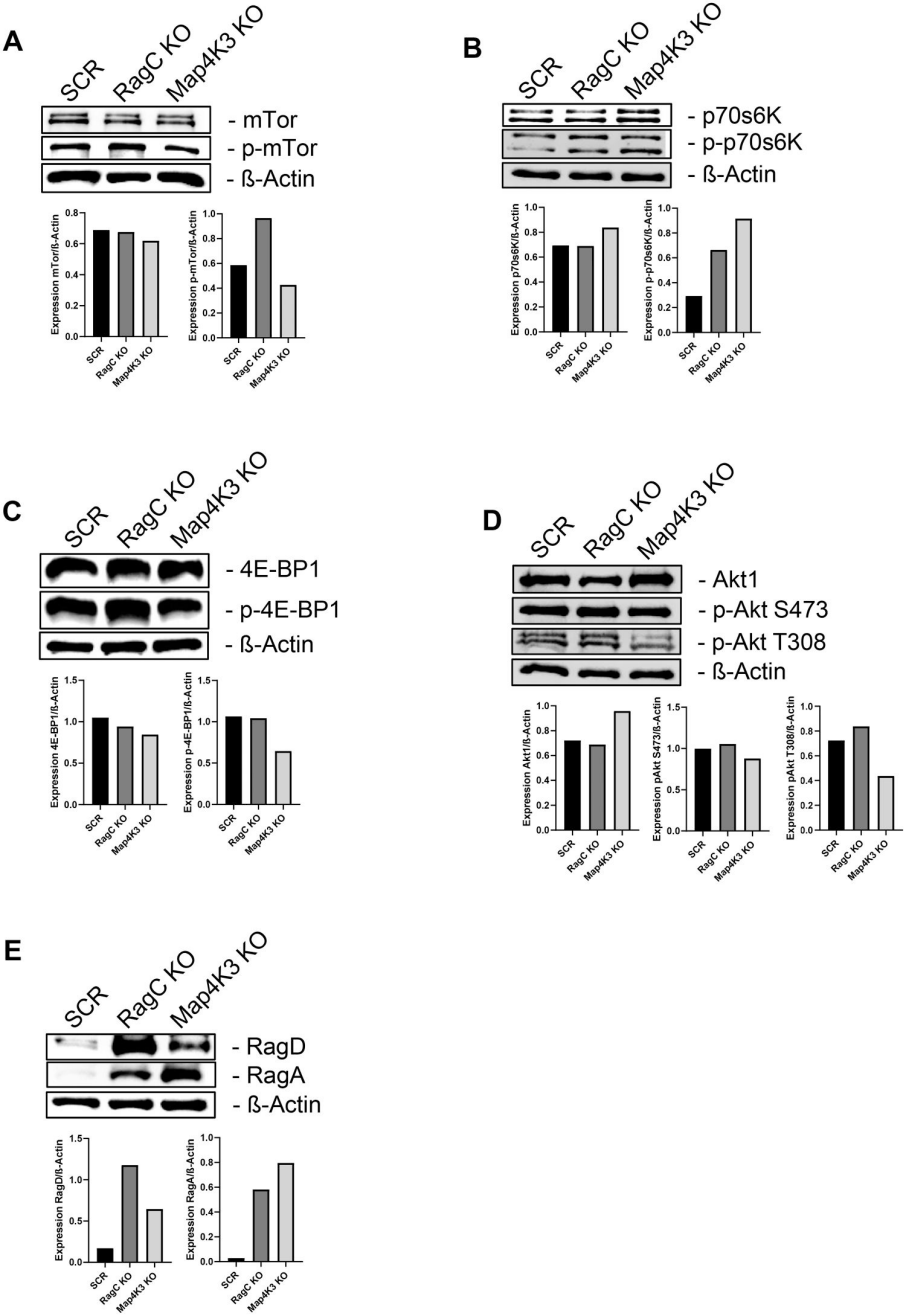
mTOR signaling in RagC- and Map4K3-deficient glioma cells. Western blot analyses for mTOR (A), p70S6K (B), 4E-BP1 (C), AKT1 (D), RagD and RagA (E). U87 KO cells were cultured in complete media (DMEM). The ratio of investigated antibodies to β-actin was determined by densitometry using ImageJ. Abbreviation: DMEM, Dulbecco’s modified Eagle’s medium.

Given that both RagC and Map4K3 are important components of the mTOR complex, we first focused on mTORC1 (Figure 3A), and the downstream p70S6 kinase and its phosphorylation (Figure 3B). Both RagC-KO and Map4K3-KO U87 cells showed significantly increased phosphorylation of p70S6K. In RagC-KO U87 cells, a correspondingly increased expression of phosphorylated mTOR was found but this was not seen in the Map4K3-KO cells. A correspondingly increased expression of phosphorylated mTOR was found only in RagC-KO U87 cells. Another downstream molecule of mTORC1, 4E-BP1 showed decreased expression (Figure 3C).

We studied the upstream region, Akt1 and its phosphorylation at serine 473 and threonine 308 (Figure 3D). There was no change in the expression of serine 473 in either RagC-KO or Map4K3-KO cells but there was a reduction at threonine 308 for Map4K3-KO cells. Figure 3E shows an increase of RagA and RagD in RagC-KO and also in Map4K3-KO cells.

There was a significant reduction in Rictor expression for both RagC- and Map4K3-deficient U87 cells, whereas Raptor remained unaffected (Figure S3A). Furthermore, p44/42-MAPK showed reduced phosphorylation in Map4K3-KO compared to SCR control and RagC-KO (Figure S3B). Similar to the increased expression of RagD in U87-KO cells, we also found increased expression of RagD in RagC-deficient U138 cells, MCF-7 cells, and IOMM-Lee cells. For Map4K3 deficiency, an increase in RagD was only seen in U138 and MCF-7 cells, whereas IOMM-Lee cells showed a decrease in RagD (Figure S3C). RagA expression in the abovementioned cell lines showed a decrease under RagC deficiency and a similar level to the control for U138 and MCF-7 cells under Map4K3 deficiency (Figure S3C). IOMM-Lee cells with Map4K3 deficiency, on the other hand, showed increased expression of RagA.

### Leucine depletion reduces proliferation only in Map4K3-KO glioma cells

**Figure 4.**
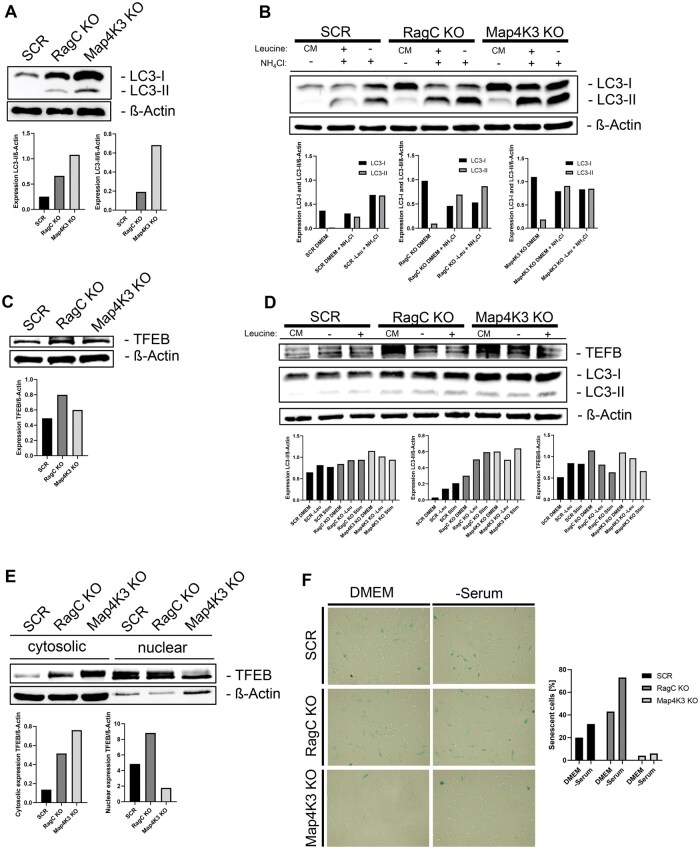
Knockout of Map4K3 and RagC leads to autophagy induction. (A) Autophagy induction of U87 RagC-KO and Map4K3-KO compared to SCR control cells via Western blot analysis. (B) Autophagy flux representing as Western blots in U87 KO cells. U87 KO cells were cultured in complete media (Leucine: CM) or in condition of amino acid deprivation (Leucine: −, 4 h), and remained untreated (NH_4_Cl: −) or were treated (NH_4_Cl: +, 4 h) with ammonium chloride (NH_4_Cl). (C) Western blot analysis of total TFEB for RagC-KO and Map4K3-KO compared with SCR control. (D) Separation of cytoplasm and nucleus showing TFEB expression for RagC-KO and Map4K3-KO compared with SCR control. (E) Western blot analysis of total TFEB and LC3 in condition of complete media (Leucine: CM) or amino acid deprivation (Leucine: −, 4 h), and restimulation with leucine-free medium including leucine according to DMEM (leucine: +, 1 h). The ratio of investigated antibodies to β-actin was determined by densitometry using ImageJ. (F) β-Gal staining of U87 RagC-KO and Map4K3-KO compared with SCR control. Quantification of green stained cells was determined using ImageJ. *n* > 100 cells per genotype.

To test whether Map4K3 or RagC respond differentially on amino acid depletion, we generated conditions of deprivation of the essential amino acid leucine. For this purpose, we assessed proliferation with the CCK-8 assay (Figure 4A). Under leucine depletion, U87 SCR controls showed a nonsignificant decrease in proliferation and appeared also not significant in RagC deficiency. In contrast, Map4K3-deficient U87 cells showed a significant proliferation decrease under leucine deprivation. Under conditions of leucine-free medium containing supplemental leucine according to control medium, U87 SCR controls and RagC-deficient U87 cells showed increased proliferation above baseline, whereas Map4K3-deficient U87 cells nearly reached baseline. Protein expression analysis was used to describe leucine deprivation in both RagC- and Map4K3-deficient U87 cells. Figure 4B shows mTOR and its phosphorylation with an increase in RagC-KO and a decrease in Map4K3-KO. Under restimulation with leucine-free medium and added leucine according to control medium, a level similar to baseline was seen. However, p70S6K showed reduced phosphorylation in Map4K3-KO cells under leucine deprivation conditions, whereas RagC-KO and SCR control showed identical expression compared with control medium (Figure 4B). 4E-BP1 showed unaltered phosphorylation for SCR control and both genotypes (Figure 4B). In contrast, the unphosphorylated moiety showed a decrease under leucine-free medium, which did not recover to baseline levels even under restimulation. Both Map4K3-KO and RagC-KO U87 cells show consistently increased expression of RagA and RagD compared to SCR controls under complete media (Figure 4C). Under leucine-free conditions, there was significant reduction in RagA without rapid recovery in RagC-KO, whereas Map4K3-KO responds with at most a small increase. SCR control remained at a constant low level under all conditions. Both RagC-KO and Map4K3-KO revealed a small increase for RagD under leucine deprivation, whereas SCR control remained unaffected. Under restimulation, only SCR control showed a decrease, RagC-KO and Map4K3-KO returned to baseline levels. AKT1 and its phosphorylation S473 and T308, respectively, are shown in Figure 4D. Both pAKT-S473 and pAKT-T308 show a slight increase under leucine-free conditions for RagC-KO and SCR control. Map4K3-KO shows an increase only for p-Akt S473 but not for p-Akt T308. Under restimulation, the same expression was seen as in control medium.

Finally, we investigated the effect of leucine deficiency on the motility of RagC- or Map4K3-deficient U87 cells (Figure 4E). Only SCR control cells and Map4K3-deficient U87 cells showed significantly reduced motility under leucine deprivation, while RagC-deficient U87 cells showed a nonsignificant reduced motility.

### Knockout of Map4K3 and RagC induces autophagy and changes cellular senescence

**Figure 5.**
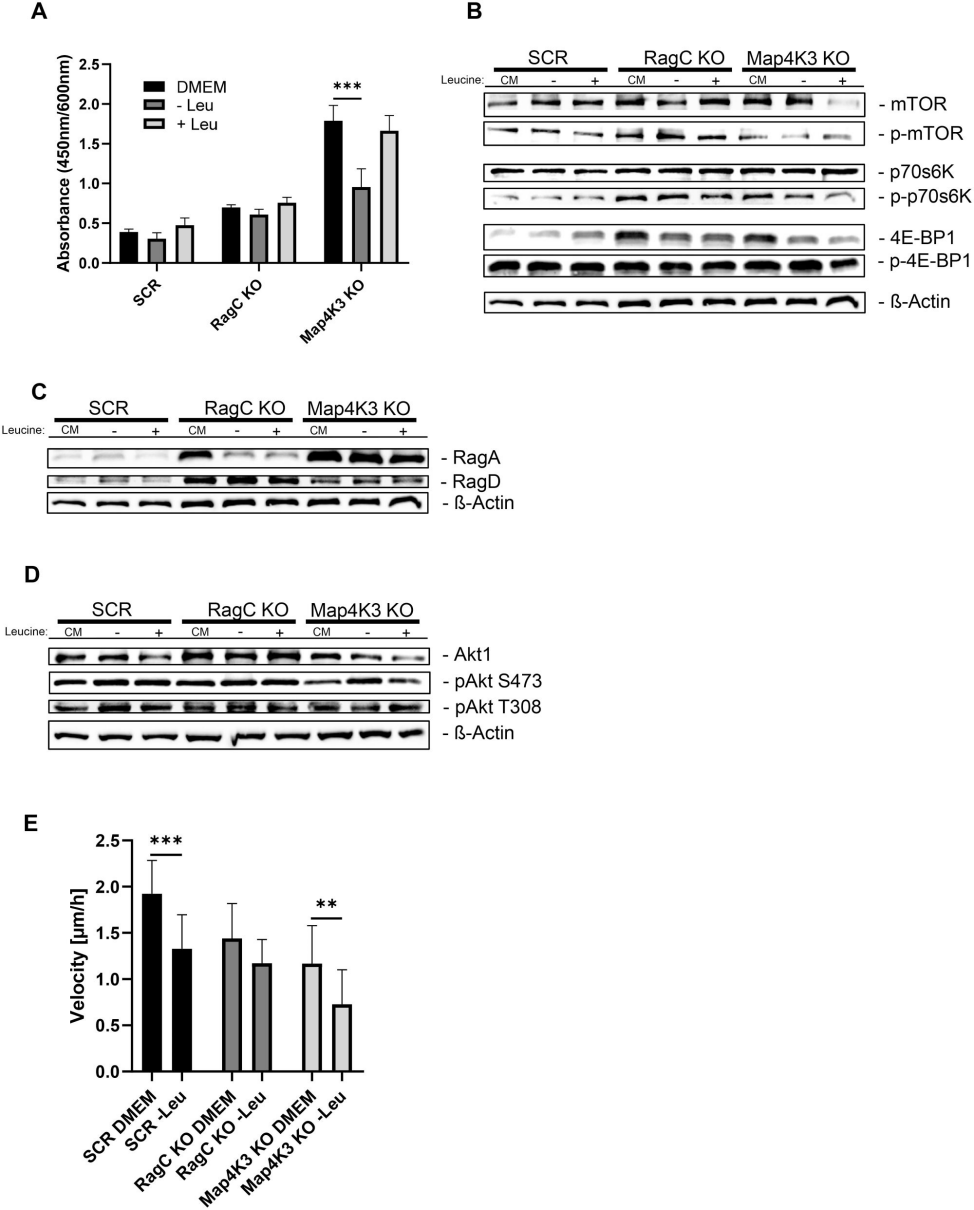
RagC- and Map4K3-deficient glioma cells under leucine-depleted condition. CCK-8 assay (A) and Western blots with mTOR, p70S6K, 4E-BP1 (B). Western Blot analysis for RagD and RagA (C) and Akt1 (D) for U87 SCR control, RagC-KO, and Map4K3 in condition of complete media (DMEM) or amino acid deprivation (−Leu, 4 h), and restimulation with leucine-free medium including leucine according to DMEM (+Leu, 1 h). (E) Observation of U87 SCR, RagC-KO, and Map4K3-KO cells over 4 h with live cell imaging and tracking of 10 cells each in condition of leucine-free medium. The ratio of investigated antibodies to β-actin was determined by densitometry using ImageJ. **P < .01, ***P < .001. Abbreviation: DMEM, Dulbecco’s modified Eagle’s medium.

To assess autophagy, we used protein expression analysis for LC3 for both RagC- or Map4K3-deficient U87 cells (Figure 5A). On one hand, we found an increased amount of free LC3 in the cytosol (equivalent to LC3-I), but also an increased amount of priority autophagosome-bound LC3 (equivalent to LC3-II). For a more detailed assessment of autophagy flux, we analyzed the amount of LC3 after treatment with ammonium chloride, which blocks lysosomal degradation of autophagosomes (Figure 5B). We found a further increase in the amount of LC3-II, which is indicative of the induction of autophagy and proper autophagosome-lysosome fusion. In addition, we found that leucine deprivation caused an even greater autophagy flux. It was thus reasonable to find out if TFEB signaling, which is mainly controlled by Map4K3 and indirectly controlled by RagC played a role for this observed increased autophagy induction. Therefore, we performed a Western blot for total TFEB and found elevated levels in RagC- and Map4K3-deficient U87 cells compared to U87 SCR controls (Figure 5C). In addition, we were interested in the effect of a leucine-deficient state on LC3 and total TFEB levels (Figure 5D). Considering LC3-II, RagC deficiency and SCR control showed strong autophagy with 4 h of leucine deprivation, whereas Map4K3-deficient U87 cells responded with reduced autophagy. Subsequent 1-h stimulation with leucine-free medium and supplemental leucine according to control medium showed induction of autophagy beyond baseline in SCR control and RagC-deficient U87 cells but not under Map4K3 deficiency. Under leucine-free conditions, TFEB showed increased expression in SCR control U87 cells, whereas RagC- and Map4K3-deficient U87 cells showed decreased expression. This decrease continued even under restimulation, whereas in U87 SCR controls it did not revert to baseline levels. Because it is known that TFEB acts as a transcription factor for autophagy in the nucleus, we performed a TFEB Western blot of a cytosolic–nuclear separation (Figure 5E). TFEB showed a markedly increased expression under RagC deficiency and even more markedly for Map4K3-deficient U87 cells in the cytoplasm compared to U87 SCR controls. In the nucleus, however, there was decreased expression of TFEB under Map4K3 deficiency compared to RagC-deficient U87 cells and U87 SCR controls. Notably, nuclear expression of TFEB under RagC deficiency was strongly increased compared to U87 SCR controls. The autophagy indicator LC3 was also examined for RagC- or Map4K3-deficient U138, MCF-7, and IOMM cells (Figure S5A). Only RagC- and Map4K3-deficient U138 cells showed an increase in LC3 expression. In contrast, MCF-7 and IOMM-Lee cells deficient in RagC or Map4K3 did not show LC3 expression.

Because senescence is another potential mTOR signaling pathway-regulated mechanism in tumor cells, we were interested in whether downregulation of RagC or Map4K3 affects senescence. Surprisingly, RagC-deficient U87 cells showed increased senescence that was even greater under serum-free conditions. By contrast, Map4K3-deficient U87 cells showed reduced senescence even under serum-free conditions.

## DISCUSSION

We aimed to examine in detail possible roles of the amino acid sensors RagC and Map4K3 in the growth and spread of gliomas, particularly high-grade gliomas. Malignant gliomas are known for their rapid growth and increased consumption of nutrients, especially amino acids such as leucine.^5^^,^^9^ Leucine represents an important stimulator of RagC and Map4K3, whose importance for mTORC1 activity and maintenance of cell growth is well described.^13^^,^^15^^,^^20^

Altered amino acid sensors have been reported in different cancers, including glioblastoma, with inconsistent results.^27^^,^^28^ RagC and Map4K3 are described to drive mTORC1 activity and a lack of them led to reduced growth and changed morphology.^12^^,^^21^ Surprisingly, we observed that RagC and Map4K3 levels are lower in high-grade gliomas compared to low-grade IDH-mutated astrocytomas. This downregulation of RagC or Map4K3 might represent a unique feature of malignant gliomas and could help to explain their growth characteristics, possibly driven by a nutritionally depleted environment.^35^^,^^36^ Furthermore, alterations in cell morphology might facilitate cell motility whereby smaller sizes gives advantage to infiltrate brain tissue. Alternatively, increased cell size led to better cell contact and improved microenvironment. Knockdown of RagC and Map4K3 changed morphology without increasing motility or altering migration behavior. The observed reduction in cell area in U87 RagC-deficient cells did not appear to have a motility-enhancing effect on glioma-specific migration. In contrast, knockdown of Map4K3 led to significant morphological changes resulting in reduced motility. It is possible that the reduced motility leads to better cell contact behavior. However, the limited value of U87 and U138 glioma cells for understanding in situ glioma cell growth and cell size/shape adjustment must be considered. The reduced Rictor expression found in this context does not rule out the possibility that mTORC2 is affected. Considering the mTORC2-dependent phosphorylation of AKT, Map4K3 in particular appears to have a possible influence on mTORC2 activity.^37^ On the other hand, the observed overexpression of p-p70S6K could result in negative feedback on the mTORC2 signaling pathway.^38^

Contrary to our expectations and most surprising, there was a proliferation-facilitating effect of U87-deficient RagC or Map4K3 cells. The increase in proliferation in the absence of RagC appears to be a general phenomenon, whereas the absence of Map4K3 leads to increased proliferation particularly for glioma cells. Notably, increased proliferation was previously reported for U2OS and HEK293 Map4K3-depleted cells.^37^ Given this cell-type specific effects, the microenvironment and the general nutrient supply of the tumor entities could play a significant role.^39^ In addition, we found expression changes among the RagGTPases. In particular, RagD appears to be a crucial initiator for the excessive proliferation.^40^ Our results are consistent with studies on increased RagD expression and are confirmed by the increased expression of RagD in all of our RagC-deficient cell lines. Since RagD is known to influence lysosomal recruitment, this could be a possible explanation for the increased proliferation in addition to the increased TFEB activity.^40^^,^^41^ Whether TFEB itself has a significant influence on proliferation remains uncertain. In addition to RagC in the RagGTPase complex, Map4K3 ensures phosphorylation of TFEB and thus regulation of autophagy.^25^ We hypothesize that the increase in proliferation is not primarily due to activation of the RagGTPase complex, but rather the product of induced autophagy under the loss of Map4K3. It is striking that the strong proliferation increase for Map4K3-deficient U87 cells was accompanied by an increased expression of RagD and LC3, which was not found for Map4K3-deficient MCF-7 or IOMM-Lee cells. Considering the insufficient autophagy in Map4K3-deficient U138 cells, RagD seems to be the main driver of proliferation. A possible explanation for the insufficient autophagy in Map4K3-deficient U138 cells might be the reduced cell size and thus limited component capacity. Unusual in this context is the increased expression of RagA, which was only found in RagC- and Map4K3-deficient U87 cells. In the other cell lines with RagC deficiency, there was a reduction, which does not suggest a compensatory increase in RagA expression, but rather implies a codependency.^12^ RagA could represent a binding site between Map4K3 and the mTOR signaling pathway and should be considered in more detail with regard to autophagy.

Since insufficient autophagy can also cause senescence, we investigated this in the absence of RagC or Map4K3 in U87 cells.^42^ The observed increased TFEB expression in RagC-deficient U87 cells without significant induction of autophagy could explain the observed cellular senescence. We also suspect that the cell size and the available cell components have an influence. The morphological change caused by the RagC-KO presumably disfavors autophagy and leads faster and earlier to senescence.^43^ In contrast, the structural cell components in the enlarged Map4K3-deficient U87 cells appear to have sufficient capacity so that no senescence was induced even after 24 h of serum-free medium. Furthermore, our leucine deprivation experiments showed that RagC-deficient U87 cells responded with a drastic decrease in RagA expression without impaired proliferation or phosphorylation of p70S6K or mTOR. This finding could support the idea that the increase in proliferation is solely due to the increased expression of RagD.^40^ In addition, the stabilizing effect on RagA seems to be absent due to the absence of RagC, since the expression of RagA does not increase under restimulation with leucine.^12^ Considering the induced senescence, a lack of suppression by RagA would be questionable in this context. In contrast, Map4K3-deficient U87 cells showed a significant reduction in proliferation with reduced phosphorylation of p70S6K or mTOR.

Taken together, the altered RagGTPase composition in gliomas could provide an explanation for the lack of efficacy in the clinical treatment of gliomas with agents such as Temsirolimus.^30^ In addition, altered autophagy and/or senescence in response to nutrient availability as a modulating aspect for glioma cell growth and infiltration needs to be recognized more in detail, and suggestions for dietary amino acid restriction should also be viewed critically in the treatment of gliomas.^44^

## Supplementary Material

nlag010_Supplementary_Data
