# Supplementary material for: RagC and Map4K3 deficiency in high-grade gliomas drives proliferation and modulates mTORC1-dependent cellular functions
Source: J Neuropathol Exp Neurol. 2026 Mar 22;85(7):777–88. doi: 10.1093/jnen/nlag010 (PMC13293255; doi:10.1093/jnen/nlag010)
Supplement: nlag010_Supplementary_Data [file nlag010_supplementary_data.zip › Kahr et al. Figure-S4.pptx]

## Slide 1
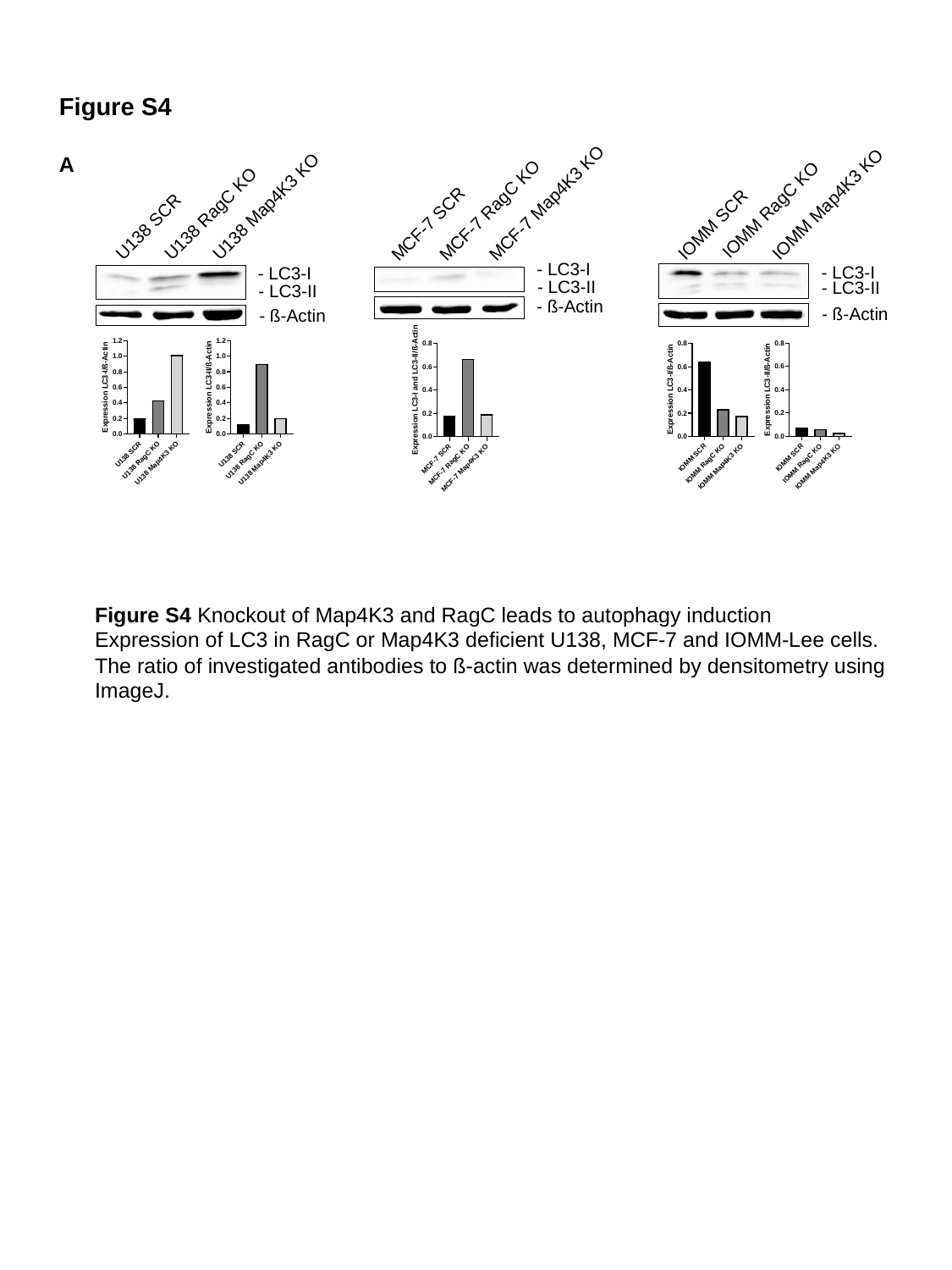

Figure S4
A
MCF-7 Map4K3 KO
IOMM Map4K3 KO
U138 Map4K3 KO
MCF-7 RagC KO
IOMM RagC KO
U138 RagC KO
MCF-7 SCR
IOMM SCR
U138 SCR
- LC3-I
- LC3-I
- LC3-I
- LC3-II
- LC3-II
- LC3-II
- ß-Actin
- ß-Actin
- ß-Actin
Figure S4 Knockout of Map4K3 and RagC leads to autophagy induction
Expression of LC3 in RagC or Map4K3 deficient U138, MCF-7 and IOMM-Lee cells. The ratio of investigated antibodies to ß-actin was determined by densitometry using ImageJ.
