# Supplementary material for: RagC and Map4K3 deficiency in high-grade gliomas drives proliferation and modulates mTORC1-dependent cellular functions
Source: J Neuropathol Exp Neurol. 2026 Mar 22;85(7):777–88. doi: 10.1093/jnen/nlag010 (PMC13293255; doi:10.1093/jnen/nlag010)
Supplement: nlag010_Supplementary_Data [file nlag010_supplementary_data.zip › Kahr et al. Figure-S1.pptx]

## Slide 1
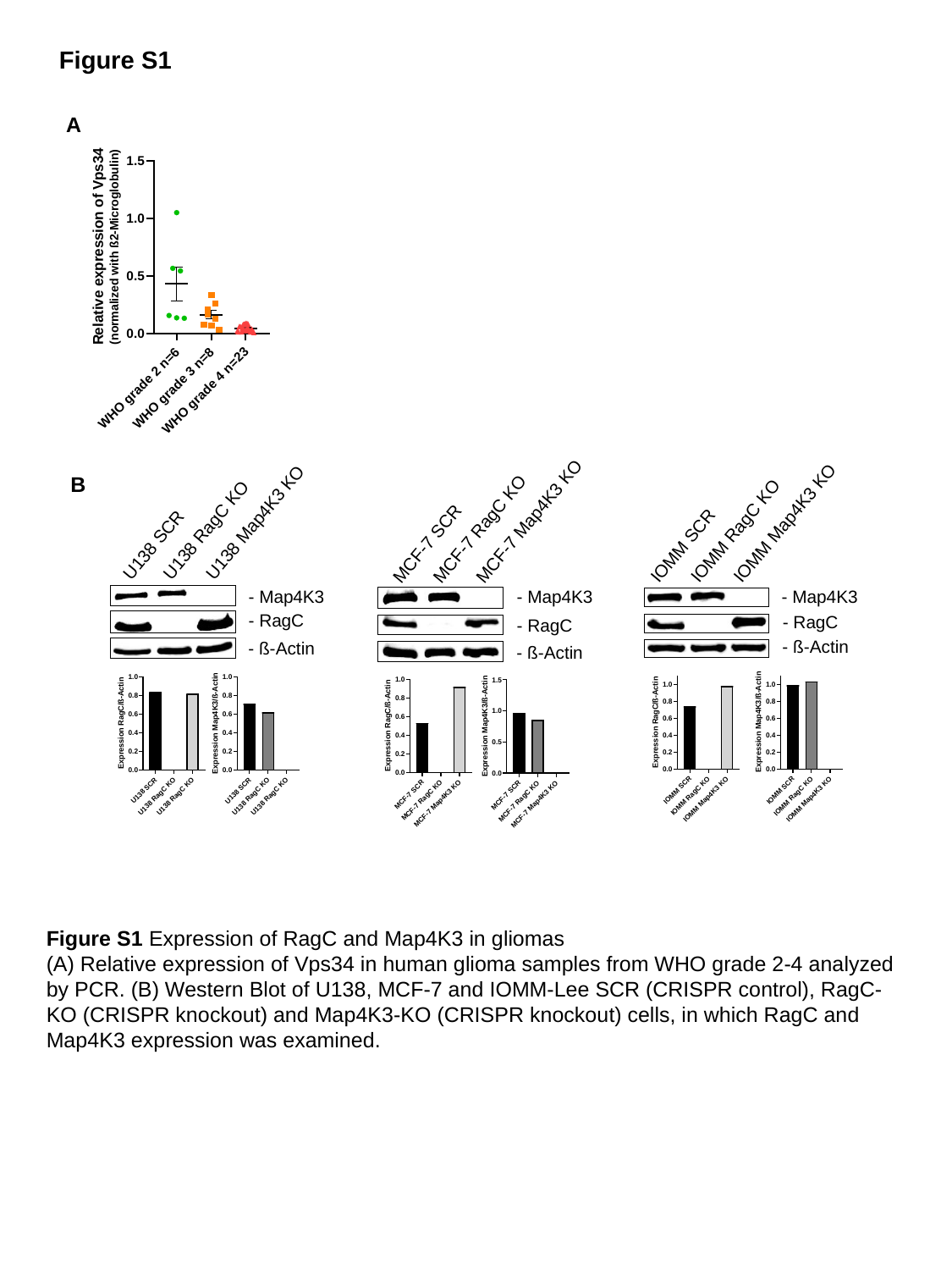

Figure S1
A
B
MCF-7 Map4K3 KO
IOMM Map4K3 KO
U138 Map4K3 KO
MCF-7 RagC KO
IOMM RagC KO
U138 RagC KO
MCF-7 SCR
U138 SCR
IOMM SCR
- Map4K3
- Map4K3
- Map4K3
- RagC
- RagC
- RagC
- ß-Actin
- ß-Actin
- ß-Actin
Figure S1 Expression of RagC and Map4K3 in gliomas
(A) Relative expression of Vps34 in human glioma samples from WHO grade 2-4 analyzed by PCR. (B) Western Blot of U138, MCF-7 and IOMM-Lee SCR (CRISPR control), RagC-KO (CRISPR knockout) and Map4K3-KO (CRISPR knockout) cells, in which RagC and Map4K3 expression was examined.
