# Supplementary material for: RagC and Map4K3 deficiency in high-grade gliomas drives proliferation and modulates mTORC1-dependent cellular functions
Source: J Neuropathol Exp Neurol. 2026 Mar 22;85(7):777–88. doi: 10.1093/jnen/nlag010 (PMC13293255; doi:10.1093/jnen/nlag010)
Supplement: nlag010_Supplementary_Data [file nlag010_supplementary_data.zip › Kahr et al. Figure-S2.pptx]

## Slide 1
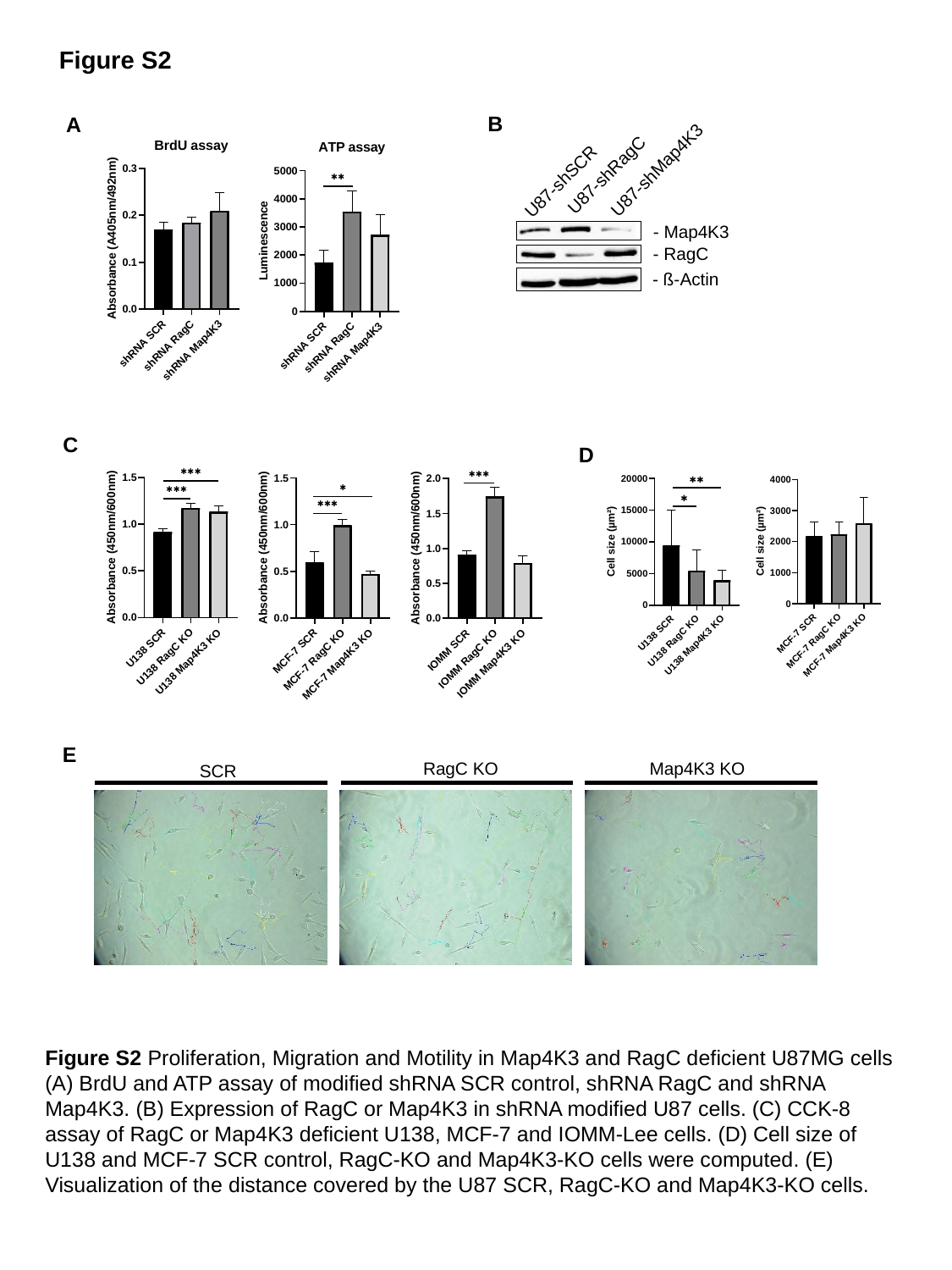

Figure S2
B
A
U87-shRagC
U87-shMap4K3
U87-shSCR
- Map4K3
- RagC
- ß-Actin
C
D
E
Map4K3 KO
RagC KO
SCR
Figure S2 Proliferation, Migration and Motility in Map4K3 and RagC deficient U87MG cells
(A) BrdU and ATP assay of modified shRNA SCR control, shRNA RagC and shRNA Map4K3. (B) Expression of RagC or Map4K3 in shRNA modified U87 cells. (C) CCK-8 assay of RagC or Map4K3 deficient U138, MCF-7 and IOMM-Lee cells. (D) Cell size of U138 and MCF-7 SCR control, RagC-KO and Map4K3-KO cells were computed. (E) Visualization of the distance covered by the U87 SCR, RagC-KO and Map4K3-KO cells.
