# Supplementary material for: RagC and Map4K3 deficiency in high-grade gliomas drives proliferation and modulates mTORC1-dependent cellular functions
Source: J Neuropathol Exp Neurol. 2026 Mar 22;85(7):777–88. doi: 10.1093/jnen/nlag010 (PMC13293255; doi:10.1093/jnen/nlag010)
Supplement: nlag010_Supplementary_Data [file nlag010_supplementary_data.zip › Kahr et al. Figure-S3.pptx]

## Slide 1
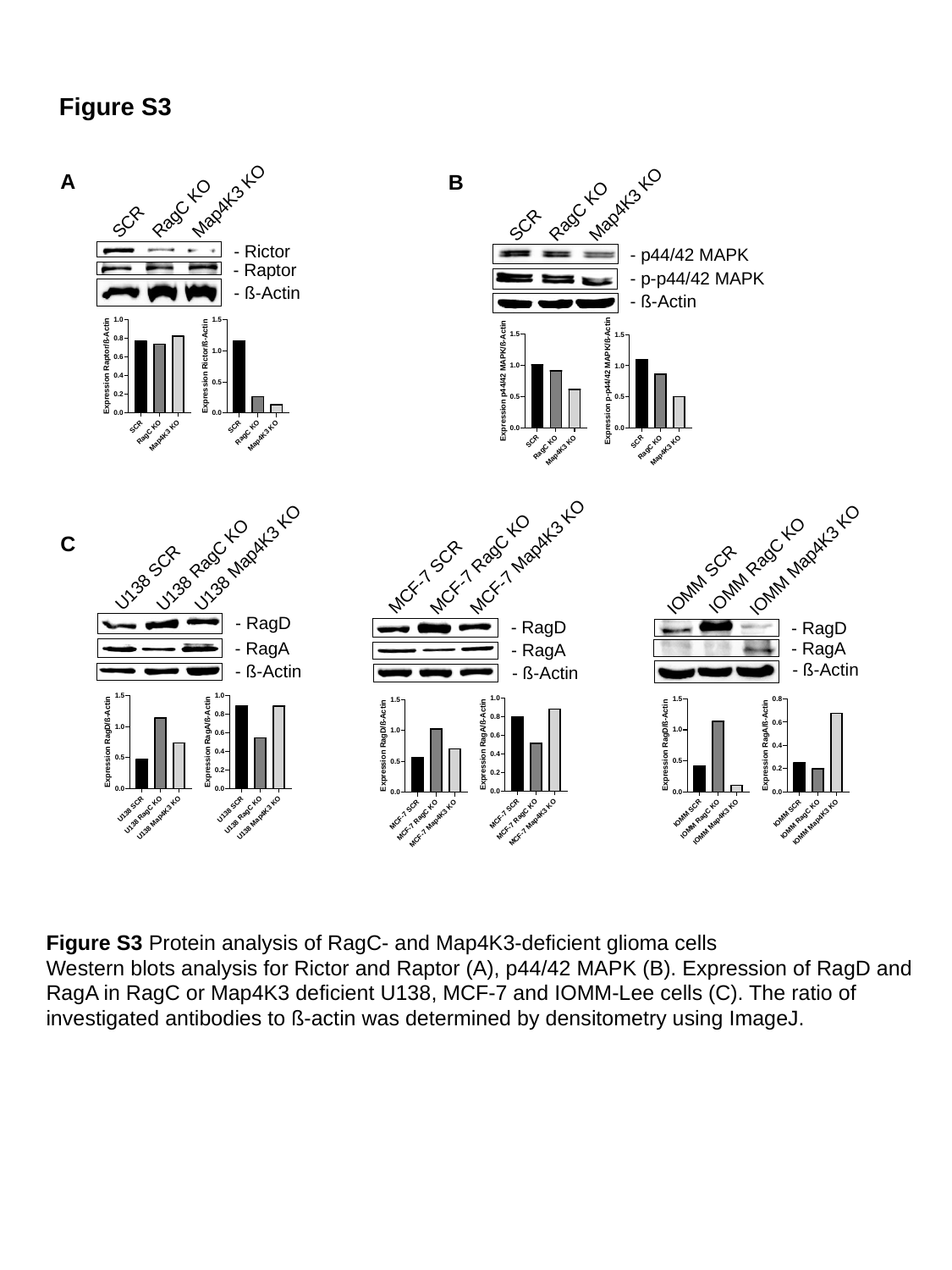

Figure S3
A
B
Map4K3 KO
Map4K3 KO
RagC KO
RagC KO
SCR
SCR
- Rictor
- p44/42 MAPK
- Raptor
- p-p44/42 MAPK
- ß-Actin
- ß-Actin
C
MCF-7 Map4K3 KO
U138 Map4K3 KO
IOMM Map4K3 KO
MCF-7 RagC KO
IOMM RagC KO
U138 RagC KO
MCF-7 SCR
U138 SCR
IOMM SCR
- RagD
- RagD
- RagD
- RagA
- RagA
- RagA
- ß-Actin
- ß-Actin
- ß-Actin
Figure S3 Protein analysis of RagC- and Map4K3-deficient glioma cells
Western blots analysis for Rictor and Raptor (A), p44/42 MAPK (B). Expression of RagD and RagA in RagC or Map4K3 deficient U138, MCF-7 and IOMM-Lee cells (C). The ratio of investigated antibodies to ß-actin was determined by densitometry using ImageJ.
